# Comparative Study Using Different Infrared Zones of the Solventless Activation of Organic Reactions

**DOI:** 10.3390/ijms12128575

**Published:** 2011-11-29

**Authors:** María Olivia Noguez Córdova, Carlos I. Flores Ramírez, Benjamín Velasco Bejarano, Gabriel A. Arroyo Razo, Francisco J. Pérez Flores, Vladimir Carranza Tellez, René Miranda Ruvalcaba

**Affiliations:** 1 Department of Chemistry, Faculty of Superior Studies Cuautitlan, Field 1, Autonomous National University of Mexico, Av. May 1st s/n, Z. C. 54740, Cuautitlan Izcalli, State of Mexico, Mexico; E-Mails: olinoco@yahoo.com.mx (M.O.N.C.); alcor_zet@hotmail.com (C.I.F.R.); qfbbevebe@gmail.com (B.V.B.); garroyo@unam.mx (G.A.A.R.); 2 Mass Spectrometry Laboratory, Institute of Chemistry, Autonomous National University of Mexico, Outer City, University City, Z. C. 04510, Coyoacan, D. F., Mexico; E-Mail: japeflo10@hotmail.com; 3 Mass Spectrometry Laboratory, Center of Chemistry, Institute of Sciences, Benemérita University of Puebla, Z. C. 72570, Puebla, Mexico; E-Mail: vcarrant@msn.com

**Keywords:** solventless, comparative study, infrared irradiation, green approach

## Abstract

In this work, the results of a study comparing the use of irradiation from different regions of the infrared spectrum for the promotion of several organic reactions, are presented and discussed. This use of eco-conditions provides a green approach to chemical synthesis. A set of ten different organic reactions were evaluated, including the Knoevenagel, Hantzsch, Biginelli and Meldrum reactions. It is important to highlight the use of a commercial device that produces infrared irradiation in the near infrared region and its distribution by convection providing heating uniformity, significantly reducing reaction times, achieving good yields and proceeding in the absence of solvent. It is also worth noting that a variety of different reactions may be performed at the same time. Finally, the products obtained were identified using TLC, together with corresponding MS-data, complementarily in comparison of NMR ^1^H and ^13^C data with literature information.

## 1. Introduction

In recent years there has been a concerted effort to introduce everybody involved with chemistry with the subject of the 12 green chemistry principles, both in the classroom and in the laboratory [1–7] In this sense the phrase “Green Chemistry” implies reaction optimization with respect to materials and energy usage, waste reduction from all sources, and overall cost reduction. In addition, minimization of toxicity and hazards as well as maximization of safety practices must be also considered.

In relation to the sixth principle of the Green Chemistry Protocol, heating by infrared irradiation has been around a long time, and consequently its many advantages have been applied to satisfy a wide range of academic and industrial processes. New applications arise continually that take advantage of this highly efficient, controllable and rapid response heat source. Consequently, and as a part of our main chemical research interests, our research group has published several green approaches to chemical synthesis using activation by infrared irradiation, mainly from middle wavelengths, for many organic transformations [8–10].

On the other hand, there is a commercial device, “Flavor Wave^®^” (Watts 1300 W, Volteage 110V/120V-60 Hz|220V/240V-60 Hz), that combines near infrared irradiation with heat transfer by convection. This reduces heating times by increasing the efficiency and uniformity of the heating. The goal of this work is therefore a comparative study of various typical organic reactions using, on the one hand, middle wavelength infrared irradiation (*vide supra*) and, on the other and for the first time, near-infrared irradiation generated by a tungsten-halogen lamp (1.15–1.5 microns). Both reactions take place in the absence of solvent.

## 2. Results and Discussion

In Table 1 were summarized the results of a set of ten different reactions, as well as the corresponding reaction conditions. During the development of this work, the expected molecules were obtained using mainly as activating mode near infrared irradiation compared with middle wavelength infrared irradiation. For near infrared irradiation, it is worth noting the significantly reduced reaction times of this novel mode (to the half and even to a third, for example entries **g** and **h**), in comparison with the results found in the literature [8–10] using middle wavelength infrared irradiation. It is also important to highlight the possibility of carrying out several different reactions at the same time due to the design of the container providing efficiency due to the heating uniformity provided by convection that allows placing of multiple reaction flasks.

In general, the advantages of infrared-assisted reactions lie not only in their higher speed, but also because of the fact that these reactions are energetically favored, in addition to being generally cleaner and more economical than using standard conditions such as a thermal heating mantle. In addition, when infrared-assisted reactions are carried out in the absence of a solvent, the reactions become “greener”, offering environmentally friendly ways of practicing chemistry.

Furthermore, it is important to take into account interesting advantages resulting from the use of near infrared irradiation [11] as a new mode in chemical reactions; these include: the immediate response time for the heat source (<1 s), the efficient use of applied energy, favoring the product by convection due to the minimal amount of internal moving air, the long life time of the tungsten-halogen filament that is considered as a clean energy source with very low environmental impact (*vide supra*).

In regard to the identification of the molecules obtained (**3a**–**j**), they were detected at the reaction using thin layer chromatography that monitored the disappearance of the substrate (**1**) and consequently detected the presence of **3a**–**j**.

Once the products were purified, the corresponding (EIMS) data were obtained and compared with the literature [8–10] (Table 2). The characteristic peaks provide evidence for the presence of the target molecules; moreover, NMR data was also in agreement, by comparison with literature information [8], as it can see for example in three selected examples [12].

## 3. Experimental

The benzaldehyde and other reactants used as substrates are commercially available (Sigma-Aldrich Chemical Co.) and were employed without further purification. The reactions were monitored by TLC, on percolated (0.25 mm) Merck silica-gel 60-F_254_ aluminum sheets. Product visualization was carried out using a 254 and 365 nm UV lamp, I_2_ or CeSO_4_·H_2_SO_4_ 1%. EIMS were acquired on a JEOL MStation JMS-700 mass spectrometer. EIMS was performed with a source temperature of 230 °C, an ionization energy of 70 eV and an ionization trap current of 300 μA. NMR experiments were carried out using a Varian Mercury-300 at 300 MHz and 75 MHz for hydrogen and carbon respectively, the solvents used were DMSO-d6 and CDCl_3_; TMS was employed as the internal reference. The middle infrared irradiation was performed using a Phillips IR lamp (375 W/220 V) integrated to an infrared reactor^8^ designed by our research group, and validated by a wide number of applications. The Flavorwave Oven^®^ uses a tungsten-halogen lamp which produces near infrared waves and complements heat distribution by convection.

General Method. (**3a**–**j**). A mixture of benzaldehyde and the corresponding reagent (**2a**–**j**) (1 mmol of each), were thoroughly mixed in a round-bottomed flask (10 mL). These mixtures were exposed to infrared irradiation (middle and near wavelengths), respectively. The corresponding reactions were monitored by TLC using the reported eluent (middle IR). Purification of the products was carried out by means of the methodology reported for each product [8].

## 4. Conclusions

Infrared irradiation from the near region and distributed by convention is offered as a very convenient mode to activate organic reactions. It is highly efficient, controllable and clean and opens the possibility of increasing the green approach to synthetic chemistry.

## Figures and Tables

**Table 1:** Near *vs.* middle infrared irradiation for the completion of organic reactions.

Substrate	Conditions		Time	
			Literature [8]	This work
	Reagent	Product	Middle infrared/Temp	Near infrared/Temp
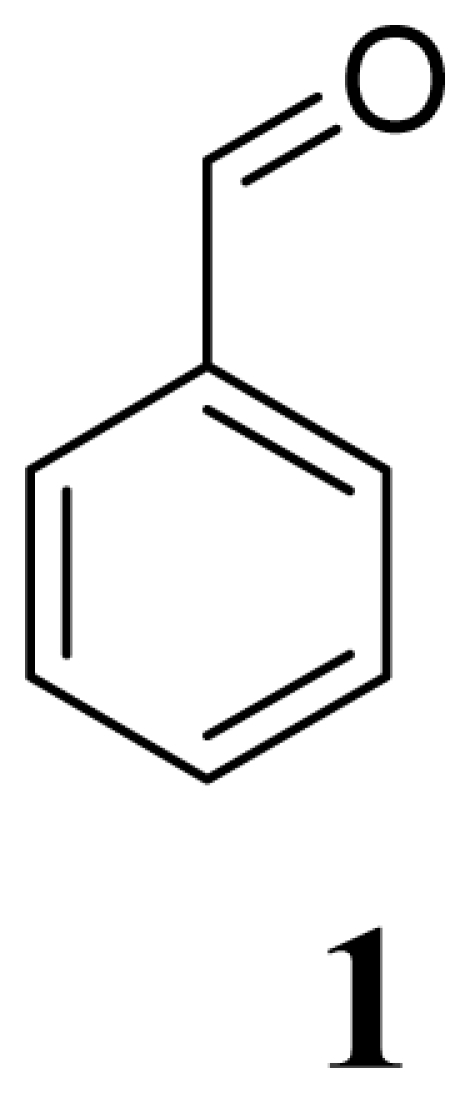	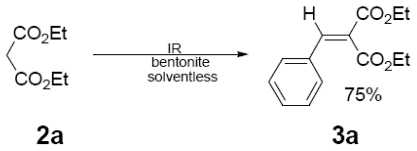		15 min/80 °C	7 min/170 °C
	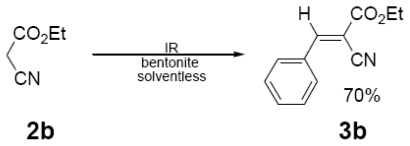		15 min/80 °C	7 min/170 °C
	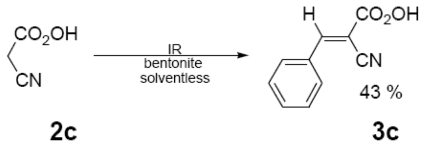		15 min/80 °C	7 min/170 °C
	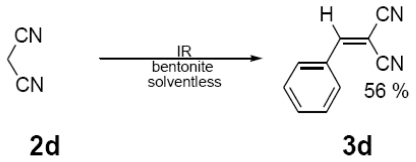		15 min/80 °C	7 min/170 °C
	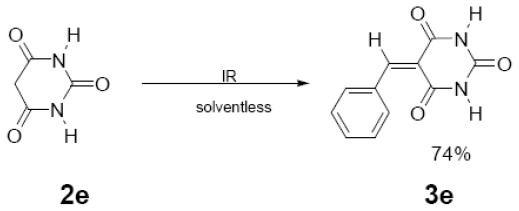		45 min/80 °C	20 min/170 °C
	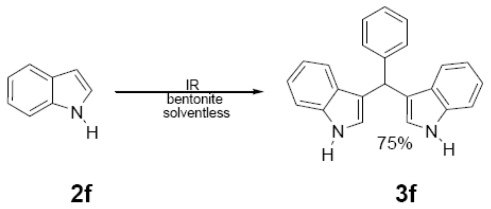		15 min/80 °C	7 min/170 °C
	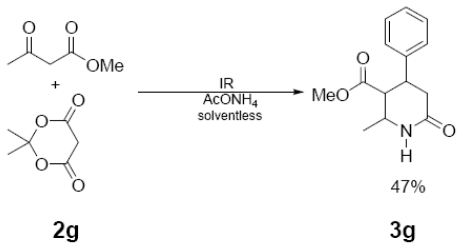		3 h/80 °C	50 min/170 °C
	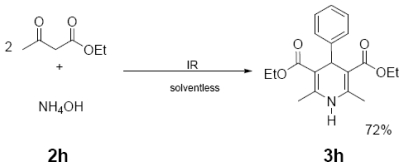		3 h/80 °C	50 min/170 °C
	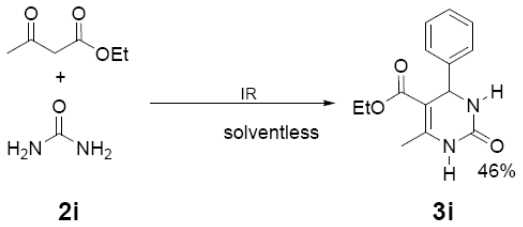		3 h/80 °C	1.5 h/170 °C
	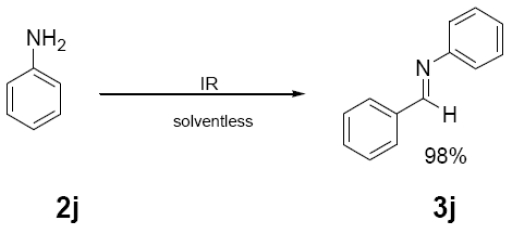		40 min/80 °C	20 min/170 °C

**Table 2:** Characteristic peaks for the target molecules, revealed by EIMS and compared with data reported in the literature.

Compound	*m*/*z* (% relative abundance) M^+•^	Others Peaks *m*/*z* (% relative abundance)
**3a**	248 (23)	176 (100), 172 (13), 77 (20)
**3b**	201 (39)	129 (99), 125 (22), 77 (18)
**3c**	173 (95)	172 (100), 156 (8),146 (5), 128 (23), 101 (8)
**3d**	154 (100)	127 (75), 100 (11)
**3e**	216 (63)	215 (100), 172 (63), 145 (7), 127 (17), 102 (26)
**3f**	322 (100)	321 (34), 245 (52)
**3g**	229 (88)	214 (35), 186 (66), 131 (100), 91 (42), 77 (39)
**3h**	329 (44)	300 (21), 284 (25), 256 (30), 252 (100)
**3i**	260 (27)	231 (66), 187 (39), 183 (100)
**3j**	181 (63)	105 (100), 77 (34)
